# Measuring Depression Severity With Clinical Global Impression–Severity Scale Scores From Clinical Notes Using Large Language Models: Validation Study

**DOI:** 10.2196/86906

**Published:** 2026-08-10

**Authors:** Kevin Li, Ayah Zirikly, Sarah C Collica, Fernando S Goes, Congwen Zhao, Trang Nguyen, Jane P Gagliardi, Benjamin A Goldstein, Hwanhee Hong, Elizabeth A Stuart, Peter P Zandi

**Affiliations:** 1 Department of Psychiatry and Behavioral Sciences, School of Medicine, Johns Hopkins University, 550 N Broadway, Baltimore, MD, 21205, United States, 1 410-614-1923; 2 Department of Computer Science, Whiting School of Engineering, Johns Hopkins University, Baltimore, MD, United States; 3 Department of Computer Science, School of Engineering and Applied Science, George Washington University, Washington, DC, United States; 4 Department of Biostatistics and Bioinformatics, School of Medicine, Duke University, Durham, NC, United States; 5 Department of Biostatistics, Bloomberg School of Public Health, Johns Hopkins University, Baltimore, MD, United States; 6 Department of Psychiatry and Behavioral Sciences, School of Medicine, Duke University, Durham, NC, United States

**Keywords:** large language models, natural language processing, psychiatry, depression, clinical global impression, clinical global impression–severity, CGI-S, measurement-based care, electronic health records, interrater reliability, artificial intelligence, AI

## Abstract

**Background:**

Real-world psychiatric care is marked by wide heterogeneity in clinical presentations and outcomes, underscoring the need for systematic approaches to outcome measurement. The Clinical Global Impression–Severity (CGI-S) scale is a brief, clinician-rated measure of overall illness severity that is widely used in psychiatric research, but rarely documented in routine care. Large language models (LLMs) may enable automated extraction of CGI-S scores from narrative clinical notes, thereby providing scalable outcome measures for real-world clinical care and research.

**Objective:**

The study aimed to evaluate whether LLMs can estimate CGI-S scores from psychiatric clinical notes for patients with major depressive disorder (MDD) and to compare performance across prompting strategies and model architectures.

**Methods:**

We extracted psychiatrist-authored notes from the Johns Hopkins electronic health record. Three board-certified psychiatrists independently rated 77 clinical notes using a validated depression-specific Clinical Global Impression (CGI) rubric. Weighted Cohen kappa coefficients were calculated to assess inter-rater reliability and model-human agreement. We evaluated GPT-4o under zero-shot and few-shot prompting conditions and Llama-4 under zero-shot prompting. Model performance was assessed by comparing LLM-generated scores to individual rater scores and consensus ratings. Exploratory analyses evaluated whether agreement varied by patient demographics, care setting, note length, or the percentage of copy-forwarded text within each note.

**Results:**

Interrater reliability among psychiatrists was high (κ=0.77‐0.78). GPT-4o with zero-shot prompting demonstrated the highest agreement with average human ratings (κ=0.85, 95% CI 0.78-0.90), and few-shot prompting did not improve performance. In contrast, Llama-4 with zero-shot prompting demonstrated lower agreement with average human ratings (κ=0.70, 95% CI 0.55‐0.80). Model agreement did not significantly differ across age, sex, race, treatment location, or the percentage of copy-forwarded text, but it was significantly lower for notes below the median note length than for notes at or above the median length (κ=0.72 vs 0.92; *P*=.003).

**Conclusions:**

LLMs can estimate clinician-rated CGI-S scores from psychiatric clinical notes for patients with MDD at a level of agreement comparable to that of expert interrater reliability. Performance varied by model architecture, with GPT-4o outperforming an open-source alternative. If further validated, this approach may support scalable outcome measurement in research settings and inform future efforts to implement measurement-based care in real-world psychiatric practice.

## Introduction

Major depressive disorder (MDD) is a leading cause of disability worldwide, accounting for approximately one-third of years lived with disability from mental disorders, and its incidence has increased by approximately 60% between 1990 and 2019 [1,2]. The clinical course and treatment response in MDD are highly variable, and many individuals experience incomplete remission or treatment resistance [3,4]. Capturing this variability in real-world settings is important to gain a more nuanced phenotypic understanding of depression to inform more personalized care. Although clinical trials routinely use validated symptom rating scales, outcome data in routine psychiatric care are inconsistently measured [5], with less than 20% of mental health providers incorporating measurement-based care (MBC) into their practice and as few as 5% using it consistently at every session [6]. Although MBC has been shown to improve outcomes and is recommended in treatment guidelines [7,8], real-world uptake has been slow due to practical and organizational obstacles [9]. Implementation programs such as the National Network of Depression Centers Mood Outcomes Program have only recently demonstrated that adoption is feasible at scale when supported by coordinated infrastructure and system-level investment [10].

In this context, the Clinical Global Impression (CGI) scale [11] represents a promising candidate for scalable, measurement-based assessment in real-world settings. The CGI is a brief, Likert-type scale rated by clinicians during patient encounters and includes both the CGI-Severity (CGI-S) scale, which rates cross-sectional illness severity from 1 (not at all ill) to 7 (among the most extremely ill patients), and the CGI-Improvement (CGI-I) scale, which assesses longitudinal change from 1 (very much improved since initiation of treatment) to 7 (very much worse since initiation of treatment). The CGI-S scale, the focus of the present work and hereafter referred to simply as CGI, has been validated in a wide range of psychiatric settings (eg, inpatient [12], outpatient [9,13], and clinical trial contexts [14]) and psychiatric conditions including mood [15-18], anxiety [19,20], and psychotic disorders [21-23]. In inpatient care, CGI scores have shown strong convergent validity with established measures such as the Health of the Nation Outcome Scales (HoNOS), Mental Health Questionnaire–14 (MHQ-14), and Depression Anxiety Stress Scales–21 (DASS-21) [12], underscoring their value as a brief and pragmatic outcome measure. In depression specifically, CGI ratings have demonstrated strong correlations with standard symptom scales (r≈0.6‐0.8), including the Beck Depression Inventory (BDI), Hamilton Depression Rating Scale (HAM-D), and Montgomery-Åsberg Depression Rating Scale (MADRS), and have shown comparable or greater sensitivity to treatment-related change across outpatient and clinical trial populations [13,24-26].

Interrater reliability for CGI has generally been reported to be in the moderate-to-excellent range across psychiatric disorders, with Cohen κ or interrater coefficient values of approximately 0.7-0.8 [23,27-29]. However, critiques have highlighted its relative lack of specificity and ambiguous response anchors, which may contribute to variability in ratings [30-32]. To address these concerns, Kadouri et al [27] developed an improved CGI (iCGI) for depression, incorporating standardized case vignettes to guide rater calibration. This approach achieved excellent interrater reliability when averaging rater scores (intraclass correlation coefficient [ICC]≈0.9) and demonstrated greater sensitivity for detecting clinical change than with the HAM-D [27].

Despite extensive validation in research and its potential for broad use across conditions, CGI scores are not routinely used or documented in clinical settings, where they could serve as a readily available outcome measure for large-scale observational studies. Instead, in typical clinical practice, details regarding symptom severity and improvement are recorded in the narrative text of clinical notes rather than quantified in a standardized way.

Advances in natural language processing (NLP) have introduced the possibility of extracting outcome measures directly from unstructured text. Previous approaches demonstrated that mood states and longitudinal treatment response could be accurately classified from outpatient psychiatric notes using rule-based NLP models [33]. More broadly, NLP methods have been shown to consistently improve the performance of predictive models created solely from structured data [34-38]. More recently, large language models (LLMs) have enabled the analysis of clinical text with a deeper understanding of linguistic context and meaning, moving beyond the keyword- or rule-based features used in previous NLP approaches [39,40], with robust performance in both psychiatric feature extraction and classification tasks [41-44]. However, recent work using LLMs (GPT-4o; OpenAI) to estimate depression severity scores from primary care notes demonstrated only modest performance, likely reflecting the limited psychiatric clinical documentation in primary care settings [45].

Recent applications within psychiatry, where notes typically contain richer descriptions of symptoms and clinical impressions, have provided encouraging evidence that LLMs can generate structured clinical outcomes from narrative text. Wiest et al [46] applied a Llama-2 model to psychiatric admission notes to classify the presence of suicidal ideation, plans, or attempts, achieving more than 85% accuracy and strong agreement with expert consensus ratings. McCoy and Perlis [47] extended this work by applying reasoning-style LLM prompts to hospital discharge summaries to estimate suicide risk, yielding model-derived hazard ratios that were predictive of subsequent suicide or accidental death, while also generating step-by-step, interpretable textual explanations for the predictions. In a related study, GPT-4 was used to translate psychiatric emergency department notes into quantitative symptom ratings across domains such as mood and cognition and demonstrated that these scores could predict hospitalization and length of stay [48].

Building on this foundation, we developed and tested a pipeline for using LLMs to generate CGI scores from the clinical notes of patients with depression. We first created a psychiatrist-annotated consensus reference and then compared different zero-shot and few-shot LLM prompting approaches to assess which showed the best alignment with clinician assessment. Through this approach, we sought to evaluate the feasibility of using LLMs to generate standardized outcome scores from unstructured psychiatric documentation, thereby bridging the gap between clinical assessment and scalable measurement for clinical use and research.

## Methods

### Study Data

This study was conducted using data from the Johns Hopkins Precision Medicine Center of Excellence in Mood Disorders registry, which was established to support large-scale clinical phenotyping and research in mood disorders. The registry includes longitudinal data from Epic (Epic Systems Corp), the electronic health record (EHR) of the Johns Hopkins Health System, on all patients with at least 1 encounter for a mood disorder (*International Statistical Classification of Diseases and Related Health Problems, 10th revision* [*ICD-10*]: F30.x, F31.x, F32.x, F33.x, F34.x, or F39*) who have been seen in the system since 2013. This was a retrospective study using routinely collected clinical data; no participants were prospectively recruited. For the current study, we extracted and analyzed a stratified random sample of clinical notes using the procedures described below.

### Clinical Note Selection and Processing

We extracted from the registry a stratified random sample of 499 psychiatrist-authored clinical notes from encounters with patients who had a primary diagnosis of MDD (*ICD-10* F32.x or F33.x) and were seen in the Department of Psychiatry and Behavioral Sciences at the Johns Hopkins Hospital (JHH) or Johns Hopkins Bayview Medical Center (JHBMC) between July 1, 2016 (when Epic implementation was completed enterprise-wide), and July 31, 2024. To capture a range of clinical severity, we randomly selected clinical notes in nearly equal proportion by sex and across 4 psychiatric care settings: inpatient, outpatient, intensive outpatient or partial day hospital, and consultation services. Each clinical note corresponded to a single clinical encounter, and for inpatient encounters for which multiple psychiatrist-authored progress notes were available, up to 2 notes per encounter were retained. The clinical notes reflected routine documentation generated at the time of care and included progress notes, consultation notes, or admission history and physical (H&P) notes, depending on the clinical setting. Notes were authored by different psychiatrists practicing across these settings and reflected the variability in documentation style encountered in real-world psychiatric care. No minimum or maximum note length criteria were imposed, and no additional manual editing or segmentation of the note text was performed. Consequently, the clinical notes could contain copy-forwarded text or templated elements as would be seen in real-world practice.

Notes were randomly sampled from the full pool of 499 extracted notes to construct 2 nonoverlapping datasets: an initial calibration set (n=70) used for rater alignment and rubric calibration, and an independent test set (N=77) used for all primary analyses. The remaining notes were not used in the present study. All clinical notes were deidentified prior to annotation and model evaluation using an automated pipeline implemented in R, leveraging the *spacyr* package for named entity recognition along with custom preprocessing scripts to remove protected health information (PHI). Human raters and language models were provided with identical deidentified note text.

### Annotation and Rater Procedures

To establish a psychiatrist consensus reference set of annotated clinical notes for model calibration and testing, 3 board-certified psychiatrists (KL, FSG, and SCC) met to review the depression-validated CGI rubric developed by Kadouri et al [27]. The 3 raters were study coauthors and were not blinded to the study objective, but all ratings were completed independently and before comparison with model outputs. Specifically, each rater independently assigned a CGI score to an initial set of notes (n=70) randomly drawn from the full dataset to calibrate the annotation procedure. The raters subsequently met to review notes with a deviation of ≥2 points between any 2 raters (10/70, 14.3% notes) and resolved discrepancies through consensus discussion. We then randomly selected a new independent set of notes (N=77) to form the test dataset. Using the *kappaSize* package in R (version 1.2; R Foundation for Statistical Computing) [49], we estimated that the test dataset would allow us to detect a Cohen κ of 0.8 with a precision of at least ±0.1 (95% CI width 0.2), assuming 3 raters and 7 ordinal CGI categories.

### Model Prompting and Output Evaluation

We developed a structured prompt for CGI scoring based on the same depression-validated CGI rubric [27], which was reviewed by the human raters (Multimedia Appendix 1). We applied this prompt to each clinical note in the calibration dataset using OpenAI’s GPT-4o (model identifier gpt-4o-2024-11-20). During the initial round of calibration, we observed that some clinical notes contained temporal dynamics (eg, “depressed for several weeks but now improving”) that were not adequately captured by the original phrasing. Because CGI-S is intended to reflect the clinician’s global impression of severity at the time of the encounter rather than over a retrospective time frame, we refined the prompt to instruct the model to prioritize the patient’s current clinical presentation. This revised version was used for all subsequent analyses with OpenAI’s GPT-4o model on the test dataset of annotated clinical notes. We evaluated model performance under two conditions: (1) zero-shot, in which the model received only the prompt and the clinical note, and (2) few-shot, in which we added 6 example notes to the prompt, 1 for each CGI score from 1 to 6, selected from the initial calibration set of 70 notes for which all 3 human raters independently agreed on the score (no note was rated as 7). No model fine-tuning or supervised training was performed. To assess cross-model generalizability, we additionally evaluated an open-source LLM, Llama (version 4; Meta Platforms Inc) Maverick, (accessed through the Azure Databricks Foundation Model API end point databricks-llama-4-maverick, corresponding to the foundation model identifier system.ai.llama-4-maverick), on the independent test dataset using the same finalized zero-shot prompt and identical deidentified clinical notes used for the GPT-4o models.

### Statistical Analysis

We first assessed interrater reliability among the 3 human raters by calculating weighted Cohen kappa coefficients for each pair of raters (KL-FSG, KL-SCC, and FSG-SCC) using the independent test set of notes, where the weighted κ statistic extends the original binary version to ordinal scales by accounting for the degree of disagreement between ratings. Conventionally, κ values between 0.6 and 0.8 are interpreted as indicating substantial agreement, and values above 0.8 indicate excellent agreement [50]. We then calculated pairwise weighted κ values comparing the CGI scores generated by GPT-4o (zero-shot and few-shot prompting) and Llama-4 (zero-shot prompting) with those assigned by each human rater individually. CIs for weighted κ values were estimated using nonparametric bootstrap resampling at the note level (5000 iterations).

To evaluate how well the LLM outputs aligned with consensus ratings from the human raters, we also compared model-generated CGI scores to reference scores defined in three ways: (1) the average score of all ratings rounded up as needed; (2) the unanimous score for notes where all 3 human raters independently assigned the same score; and (3) the majority score for notes where at least 2 of the 3 raters agreed.

Finally, we conducted exploratory analyses to assess whether the agreement between model-based scores and human ratings, as estimated by the weighted κ values, was significantly different according to the age, sex, or race of the patient; the clinical location of the encounter; the length of the clinical note; or the percentage of copy-forwarded text in the note. For comparisons by clinical location, we counted intensive outpatient or partial day hospital encounters as inpatient and consultation services encounters as outpatient. For note length, we dichotomized notes by the median word count (1007, IQR 646-1535 words). For the percentage of copy-forwarded text, we dichotomized notes by the median percentage (81.3%, IQR 65.4%-88.5%). For all comparisons, we used average scores from all 3 human raters and the results from the GPT-4o zero-shot model, which demonstrated the highest agreement with human ratings. We evaluated the significance of differences in weighted κ values between strata using a permutation procedure. Specifically, we randomly split the clinical notes into subsamples equal in size to the stratum of interest 5000 times and recalculated weighted κ values within these randomly generated substrata. The proportion of iterations in which the difference in weighted κ values was equal to or greater than the observed difference was taken as the empirical *P* value, representing the probability of obtaining the observed difference by chance. All analyses were carried out with the *irr* package in R [51].

### Ethical Considerations

This study was approved by the Johns Hopkins School of Medicine institutional review board (IRB; IRB00312852) under a waiver of consent. All analyses of the clinical notes were conducted in a HIPAA (Health Insurance Portability and Accountability Act)-compliant computational environment following Johns Hopkins institutional policies. All analyses with OpenAI’s GPT-4o model were carried out via a HIPAA-secure API with content logging and filtering disabled.

## Results

### Descriptive Statistics

Table 1 shows the demographic characteristics of the patients whose clinical notes were included in our test dataset, along with the treatment locations from which the notes were drawn.

**Table 1.** Test set patient demographic characteristics (N=77).

Characteristics	Test set, n (%)
Age (years)	
<40	43 (56)
≥40	34 (44)
Sex	
Female	43 (56)
Male	34 (44)
Race	
People of color (Black, Asian, and other)	27 (35)
White	50 (65)
Treatment location	
Inpatient	33 (43)
Outpatient	44 (57)

### Interrater and Model Agreement

**Figure 1.**
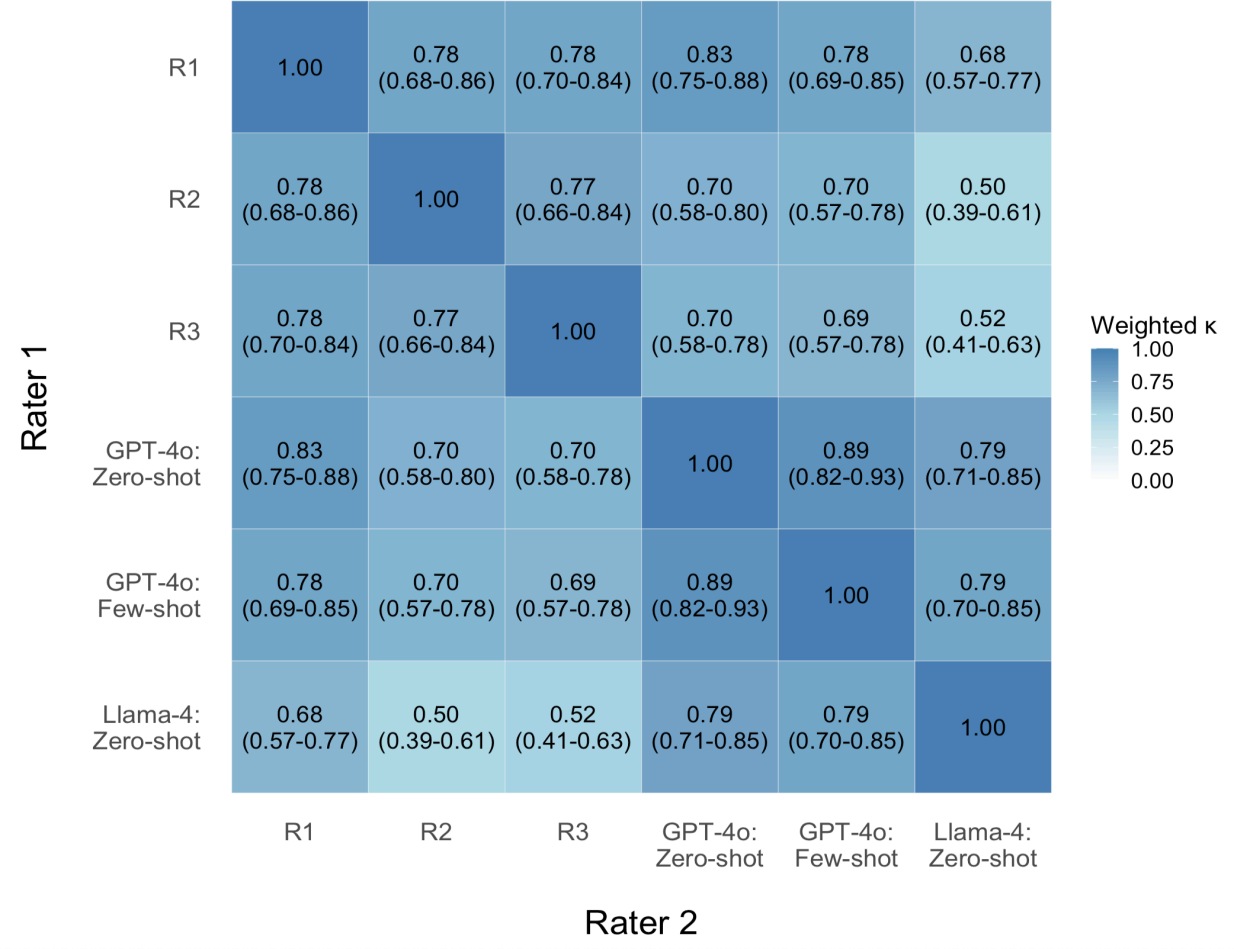
Pairwise weighted Cohen κ values between human raters and large language models (LLMs). Pairwise weighted Cohen κ values showing agreement among 3 human raters (R1, R2, and R3) and between each rater and each LLM. GPT-4o was evaluated under both zero-shot and few-shot prompting conditions, and Llama-4 was evaluated under a zero-shot prompting condition. Each cell represents agreement between a pair of raters or models. κ values between 0.40 and 0.60 indicate moderate agreement, values between 0.60 and 0.80 indicate substantial agreement, and values >0.80 indicate excellent agreement. Cells display weighted Cohen κ values; values in parentheses indicate 95% CIs estimated via nonparametric bootstrap resampling (5000 iterations).

We computed weighted κ values to assess agreement between each pair of human raters and between each model and each human rater (the full matrix is shown in Figure 1). GPT-4o was evaluated under both zero-shot and few-shot prompting strategies, and Llama-4 was evaluated under a zero-shot prompting strategy.

All 3 human rater pairs had similar interrater reliability (κ=0.77-0.78). Among model-human comparisons, the GPT-4o zero-shot model showed the highest agreement with individual raters (κ=0.83, 95% CI 0.75-0.88), followed closely by the GPT-4o few-shot model (κ=0.78, 95% CI 0.69-0.85), suggesting that additional prompting examples did not meaningfully improve agreement. The remaining GPT-4o model-human agreement pairings approached the level of human-human agreement (κ=0.70, 95% CI 0.58-0.80 vs κ=0.77, 95% CI 0.66-0.84), while Llama-4 zero-shot agreement with individual raters was more variable and lower than that of the GPT-4o models: κ=0.68 (95% CI 0.57‐0.77) with R1, κ=0.50 (95% CI 0.39‐0.61) with R2, and κ=0.52 (95% CI 0.41‐0.63) with R3. When comparing models directly, the GPT-4o zero-shot and few-shot models showed high mutual agreement (κ=0.89, 95% CI 0.82‐0.93), whereas Llama-4 demonstrated substantial but slightly lower agreement with GPT-4o outputs (zero-shot comparison: κ=0.79, 95% CI 0.71‐0.85; few-shot comparison: κ=0.79, 95% CI 0.70‐0.85).

### LLM Agreement With Human Consensus Ratings

We next compared model-predicted CGI scores to a reference standard based on human consensus ratings (Table 2).

Both the GPT-4o zero-shot (κ=0.85, 95% CI 0.78-0.90) and few-shot (κ=0.84, 95% CI 0.77-0.89) models showed robust agreement with the average of the human ratings, exceeding the agreement observed between either model or any individual human rater. Llama-4 zero-shot agreement with the average human rating was κ=0.70 (95% CI 0.55‐0.80), indicating moderate to substantial agreement but lower concordance than GPT-4o. Because model-human agreement is inherently constrained by the reliability of the human reference standard, we further examined performance across 2 subsets of notes: those for which all 3 raters agreed (n=22, 28.6%) and those on which at least 2 of the 3 raters agreed (n=68, 88.3%). For notes with unanimous human agreement, GPT-4o performance remained high (zero-shot: κ=0.88, 95% CI 0.77‐0.94; few-shot: κ=0.90, 95% CI 0.80‐0.96), while Llama-4 demonstrated substantial agreement (κ=0.72, 95% CI 0.53‐0.83). For notes with majority agreement, GPT-4o agreement decreased slightly (zero-shot: κ=0.79, 95% CI 0.70-0.85; few-shot: κ=0.77, 95% CI 0.66‐0.84), approaching the level of human-human reliability (κ=0.77-0.78). Llama-4 agreement in this subset was also lower, with κ=0.60 (95% CI 0.48-0.72), consistent with moderate agreement. Given that averaging across multiple ratings improves measurement reliability [52-54] and that the GPT-4o zero-shot model generally performed better than both the GPT-4o few-shot and Llama-4 models, all subsequent analyses were conducted using the GPT-4o zero-shot model and the average of the human ratings.

**Table 2.** Weighted Cohen κ between model predictions and consensus human ratings.

Compared to clinician ratings	GPT-4o: zero-shot, κ (95% CI)	GPT-4o: few-shot, κ (95% CI)	Llama-4: zero-shot, κ (95% CI)
Average rating (all notes; N=77)	0.85 (0.78-0.90)	0.84 (0.77-0.89)	0.70 (0.55‐0.80)
Unanimous rating (notes where all 3 raters agreed; n=22)	0.88 (0.77-0.94)	0.90 (0.80-0.96)	0.72 (0.53-0.83)
Majority rating (notes where ≥2 raters agreed; n=68)	0.79 (0.70-0.85)	0.77 (0.66-0.84)	0.60 (0.48-0.72)

**Figure 2.**
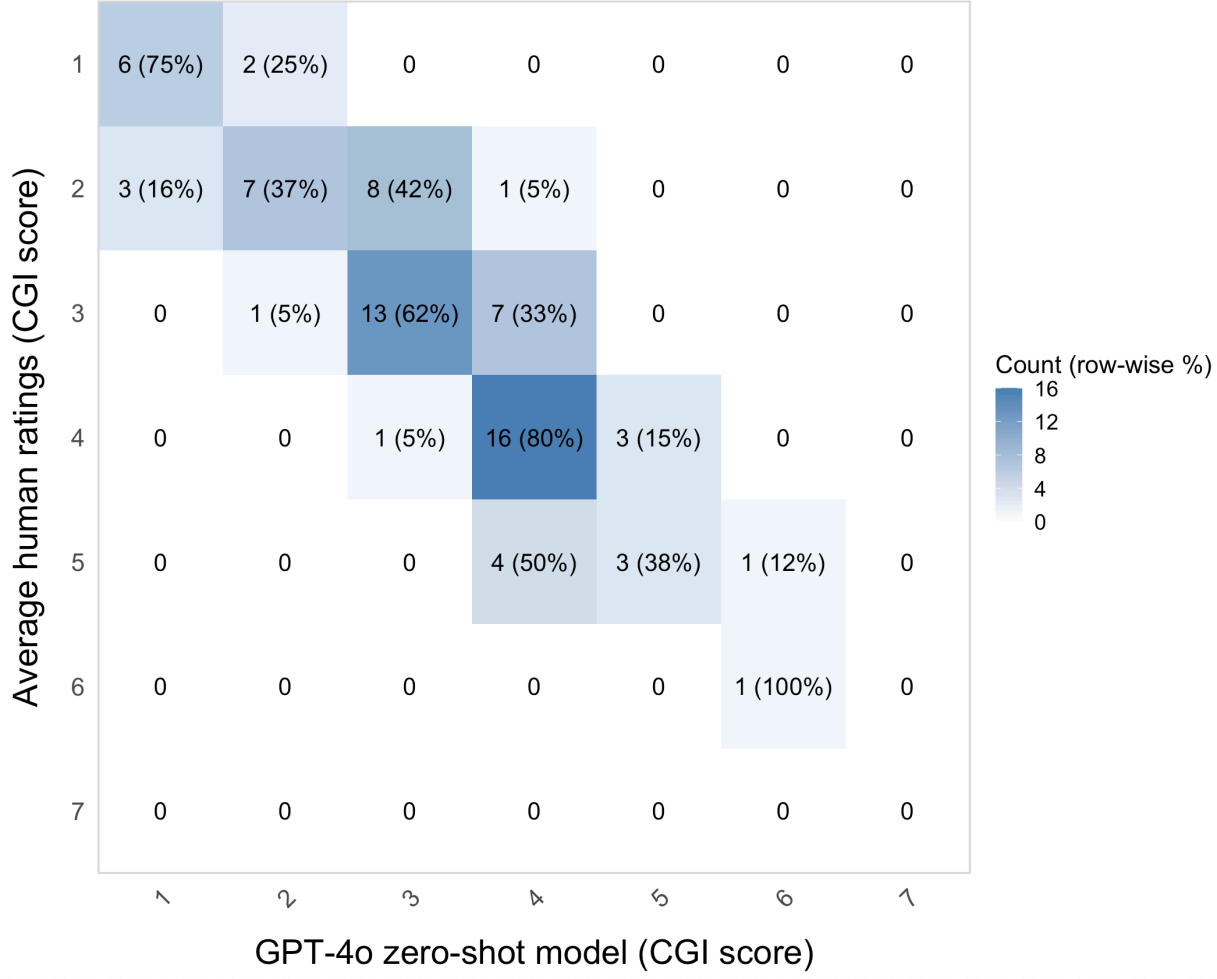
Confusion matrix comparing average human ratings with GPT-4o zero-shot model predictions. Confusion matrix showing agreement between the GPT-4o zero-shot model and the average of the human ratings for individual clinical notes. Each cell represents the number of notes assigned a given Clinical Global Impression (CGI) scale score by the model (columns) vs the average human rating (rows). Diagonal cells represent perfect agreement; off-diagonal cells indicate discrepancies, which were predominantly within 1 scale point, with a single instance of ≥2-point overestimation observed.

Figure 2 presents the confusion matrix illustrating agreement between the GPT-4o zero-shot model and the average of the human ratings for individual notes. The zero-shot model predictions were largely within ±1 CGI point of the average human rating, with no instances of ≥2-point underestimation and a single instance of ≥2-point overestimation. Among discordant cases (n=31, 40.3%), discrepancies more frequently reflected overestimation by the LLM (n=22, 28.6%) than underestimation (n=9, 11.7%). To evaluate whether directional errors were associated with patient- or note-level characteristics, we conducted exploratory univariable logistic regression analyses separately examining 2 outcomes: LLM overestimation vs all other notes and LLM underestimation vs all other notes. Covariates tested included age, sex, race, treatment location, note length dichotomized at the median, and the percentage of copy-forwarded text also dichotomized at the median. No variables were significantly associated with LLM overestimation or LLM underestimation, although the sample sizes for these comparisons were small, and caution is warranted when interpreting these findings.

To further evaluate the directional errors, we conducted an additional qualitative review of discrepant cases. This review suggested that underestimations were typically driven by notes emphasizing an unremarkable mental status examination, with only brief phrases indicating symptoms in the remainder of the note text (eg, “some instability” and “low at times”). Conversely, overestimations appeared to be driven by disproportionate emphasis on 1 or 2 specific mental status findings, which may have overshadowed a relatively less severe overall clinical assessment based on interval history or patient report. Additionally, mentions of anxiety, despite the prompt being focused on depressive symptoms, and the inpatient treatment setting were common factors associated with model overestimation.

### Subgroup Analysis

We next carried out exploratory analyses to assess whether the extent of agreement between the GPT-4o zero-shot model and the average human ratings differed according to the age, sex, or race of the patient; the clinical location; note length; or the percentage of copy-forwarded text in the note. Table 3 reports the weighted κ values for each subgroup along with the empirical significance of the difference between strata. Agreement remained high across most subgroups, although model-human agreement differed significantly by note length. Notes below the median length had lower agreement with the average human ratings than notes at or above the median length (κ=0.72 vs 0.92; *P*=.003). However, agreement did not significantly differ by the percentage of copy-forwarded text (κ=0.86 vs 0.81; *P*=.43).

**Table 3.** Weighted Cohen κ across subgroups and permutation tests for differences.

Demographic characteristics	Weighted Cohen κ	Permutation test, *P* value
Age (years)		.39
<40	0.82	
≥40	0.87	
Sex		.38
Female	0.88	
Male	0.82	
Race		.74
Non-White	0.83	
White	0.86	
Treatment location		.58
Inpatient	0.83	
Outpatient	0.86	
Word count		.003
Below median (<1007 words)	0.72	
At or above median (≥1007 words)	0.92	
Copy-forwarded text		.43
Below median (<81.3%)	0.86	
At or above median (≥81.3%)	0.81	

## Discussion

In this study, we sought to assess whether LLMs could produce reliable CGI scores, a clinician-rated measure of current patient symptom burden and impairment, from psychiatric clinical notes and how these scores compare with psychiatrist ratings. Using a prompt engineering strategy without fine-tuning, GPT-4o produced CGI scores that closely matched clinician ratings. Agreement between the model-generated and clinician-assigned CGI scores was comparable to CGI interrater reliability values previously reported among human raters (ICC or κ≈0.7-0.8) [23,27-29] and was especially high when compared with the average of all human raters (κ=0.84-0.85). Comparing the model against the average may inflate the estimate of κ, but it provides a useful indication of the upper bound of the model’s performance. Notably, the GPT-4o model performed markedly better than a leading open-source model, and few-shot prompting did not demonstrate improved agreement compared with zero-shot prompting. Although a formal statistical test comparing the zero-shot and few-shot models was not performed, the point estimates were nearly identical, with substantial overlap in their 95% CIs. These findings suggest that a clear, task-specific prompt anchored to the CGI rubric may be sufficient to achieve strong performance with GPT-4o.

Two key design choices were made to optimize model performance. First, we explicitly aligned the prompt with an established guide for CGI scoring. This design choice was intentional to standardize the rating task between the model and human raters; however, performance may not generalize to settings in which the rubric is omitted or modified. Further work is needed to evaluate whether similar performance is maintained across alternative rubrics or psychiatric conditions other than depression. We also included language instructing the model to prioritize recent information in the clinical notes, to ensure that the generated scores reflected the patient’s current status rather than past clinical history. The latter may be especially important given the frequency with which clinical notes are copy-forwarded, with studies estimating that approximately half of note content is duplicated from the author’s previous note [55,56]. The phenomenon of copy-forward within the clinical notes could potentially bias the model, without appropriate remediation, toward prioritizing prior information and lead it to fail to detect more recent clinical changes. Second, for few-shot prompting, we selected examples from notes with complete human rater agreement to maximize gold-standard fidelity. The lack of incremental benefit from few-shot over zero-shot prompting, however, may reflect a ceiling effect of our rubric-based prompt. Because human interrater reliability in our sample was κ=0.77‐0.78, model-human agreement would be expected to plateau near this level, even though agreement with a consensus gold-standard reference could be higher.

To assess model performance across potential subgroups, we further carried out stratified analyses by age, sex, race, treatment location, note length, and copy-forwarded text burden. Agreement remained high across most subgroups, but it did differ significantly by note length, with notes at or above the median length having higher agreement with average human ratings than those below the median length. This suggests that the amount of available clinical context may influence model performance, with shorter notes providing fewer details from which the model can infer depression severity. Given this finding, future work with LLM-derived CGI scores may need to be paired with minimum note length or information-sufficiency thresholds and may require human review when notes are brief or clinically sparse. This interpretation is consistent with our qualitative review of discrepant cases, in which some errors appeared to arise when brief symptom descriptions or isolated mental status findings were weighed disproportionately relative to the overall clinical impression. In contrast, agreement did not significantly differ by copy-forward burden, despite the high prevalence of copy-forwarded text in the sample. Although copy-forwarded text could theoretically bias the model toward prior clinical information rather than the patient’s current status, this finding suggests that prompting the model to prioritize recent information may have helped mitigate this concern. In exploratory error analyses, we also examined whether patient- or note-level characteristics were associated with the direction of model disagreement and found that no characteristics were significantly associated with LLM overestimation or underestimation, although these analyses were limited by the modest number of discordant cases. Together, these findings suggest generally consistent model performance across the measured subgroups while underscoring the need for larger studies to evaluate potential sources of systematic error.

Our study has several important limitations. First, we demonstrated only the reliability of an LLM compared with psychiatrists in assigning CGI scores based on information available in the text of clinical notes. This approach does not consider other information available in the EHR, such as diagnostic subtypes, clinical comorbidities, and treatment history, which may contribute to the global clinical impression of a patient. Moreover, it is unclear to what extent CGI scores based on clinical notes would compare with CGI scores assigned by psychiatrists based on their direct clinical evaluation of the patients (ie, concurrent validity) or with other measures of depression-related constructs such as the PHQ-9 or MADRS (ie, convergent validity). Unfortunately, we did not have sufficient data to examine the correlations between model-based CGI scores and other concurrent measures of depression. Importantly, LLM-generated CGI scores from clinical notes are not intended to replace clinical judgment or standardized symptom scales such as the PHQ-9 or MADRS, which serve complementary roles in clinical assessment and MBC. The relationship between CGI ratings and these scales has been well established in the prior literature, reflecting related but distinct constructs, and the present study does not aim to re-evaluate the validity of the CGI or to compare these instruments. Future work will be needed to evaluate concurrent validity by comparing text-derived CGI scores with clinician-assigned CGI ratings obtained at the time of care; however, such analyses were beyond the scope of the present study. Second, the study was limited to encounters with a primary diagnosis of MDD within a single hospital system and to notes authored by psychiatrists. Consequently, the findings may not be generalizable to other diagnoses, settings, or types of clinical providers, where varying documentation styles are practiced. Third, the 3 psychiatrist raters were study coauthors and were not blinded to the overall study objective, so potential expectancy effects cannot be fully excluded despite independent annotation procedures. This is a common limitation of annotation-based studies and should be addressed in future work using external raters independent of the study team. Finally, we observed the best performance with a closed-source LLM, which raises concerns about reproducibility, version stability over time, and cost feasibility. Performance may vary across model versions due to model updates or drift, and access to proprietary models may be constrained in some research or clinical settings. Due to these limitations, more work is needed to establish the validity of using LLMs to assign CGI scores based on clinical notes, especially in comparison with the concurrent assessments of psychiatrists based on direct clinical evaluation, and to determine the generalizability of the approach to other psychiatric diagnoses and clinical settings. Our study is an important first step, and the findings demonstrate that this future work is warranted.

These findings extend prior work showing that NLP methods, especially LLMs, can be used to support patient phenotyping and outcome measurement such as depression response or remission [33], suicide risk [46,47], and dimensional symptom ratings [48]. Our study demonstrates that LLMs can generate CGI scores, a clinician-rated global measure that is validated and widely used in clinical research but rarely captured in EHRs, based on clinical notes with agreement comparable to psychiatrist ratings. Because CGI ratings have shown good interrater reliability and strong correlations with other standardized measures [9,12,13,57], the ability to generate CGI scores from narrative clinical notes offers, if further validated, the potential of a pragmatic approach to generate outcome measures suitable for real-world studies using EHR data, as well as open up the possibility of deriving such measures in real time from ambient AI technology to facilitate MBC.

## Supplementary material

10.2196/86906 Multimedia Appendix 1 Clinical Global Impression zero-shot prompt for GPT-4o.

## Abbreviations

BDI: Beck Depression Inventory
CGI: Clinical Global Impression
CGI-I: Clinical Global Impression–Improvement
CGI-S: Clinical Global Impression–Severity
DASS-21: Depression Anxiety Stress Scales–21
EHR: electronic health record
H&P: history and physical
HAM-D: Hamilton Depression Rating Scale
HIPAA: Health Insurance Portability and Accountability Act
HoNOS: Health of the Nation Outcome Scales
ICC: intraclass correlation coefficient
*ICD-10*: *International Statistical Classification of Diseases and Related Health Problems, tenth revision*
iCGI: improved Clinical Global Impression
IRB: institutional review board
JHBMC: Johns Hopkins Bayview Medical Center
JHH: Johns Hopkins Hospital
LLM: large language model
MADRS: Montgomery-Åsberg Depression Rating Scale
MBC: measurement-based care
MDD: major depressive disorder
MHQ-14: Mental Health Questionnaire–14
NLP: natural language processing
PHI: protected health information
